# Imaging Acute Stroke: From One-Size-Fit-All to Biomarkers

**DOI:** 10.3389/fneur.2021.697779

**Published:** 2021-09-23

**Authors:** Jianfei Lu, Qiyong Mei, Xianhua Hou, Anatol Manaenko, Lili Zhou, David S. Liebeskind, John H. Zhang, Yao Li, Qin Hu

**Affiliations:** ^1^Central Laboratory, Renji Hospital, Shanghai Jiao Tong University School of Medicine, Shanghai, China; ^2^Department of Neurosurgery, Changzheng Hospital, Navy Medical University, Shanghai, China; ^3^Department of Neurology, Southwest Hospital, Army Medical University, Chongqing, China; ^4^National Health Commission Key Laboratory of Diagnosis and Treatment on Brain Functional Diseases, The First Affiliated Hospital of Chongqing Medical University, Chongqing, China; ^5^Department of Neurology, Chinese People's Liberation Army General Hospital, Beijing, China; ^6^Neurovascular Imaging Research Core and University of California Los Angeles Stroke Center, University of California, Los Angeles, Los Angeles, CA, United States; ^7^Department of Anesthesiology, Loma Linda University School of Medicine, Loma Linda, CA, United States; ^8^School of Biomedical Engineering, Shanghai Jiao Tong University, Shanghai, China

**Keywords:** ischemic stroke, tissue window, metabolic imaging, molecular imaging, reperfusion therapy

## Abstract

In acute stroke management, time window has been rigidly used as a guide for decades and the reperfusion treatment is only available in the first few limited hours. Recently, imaging-based selection of patients has successfully expanded the treatment window out to 16 and even 24 h in the DEFUSE 3 and DAWN trials, respectively. Recent guidelines recommend the use of imaging techniques to guide therapeutic decision-making and expanded eligibility in acute ischemic stroke. A tissue window is proposed to replace the time window and serve as the surrogate marker for potentially salvageable tissue. This article reviews the evolution of time window, addresses the advantage of a tissue window in precision medicine for ischemic stroke, and discusses both the established and emerging techniques of neuroimaging and their roles in defining a tissue window. We also emphasize the metabolic imaging and molecular imaging of brain pathophysiology, and highlight its potential in patient selection and treatment response prediction in ischemic stroke.

## Introduction

Stroke is the worldwide leading cause of death and adult disability. More than 80% of all strokes are caused by brain ischemia, which results from obstruction of one or more cerebral arteries. Rapid and safe restoration of the blood flow through thrombolysis or/and thrombectomy is the only approved therapy for ischemic stroke. Such treatment is strictly limited by a narrow time window and need to be performed within the first few hours after the onset of symptoms (1, 2). The “time is brain” mantra has been the golden principle for acute management of ischemic stroke for decades. Due to the narrow therapeutic window and strict indications, recanalization therapy is restricted to only a small fraction (≤ 10%) of stroke patients^1^. In the past decade, accumulating clinical trials have shown that with the patients selecting by neuroimaging, the time window for reperfusion has been iteratively extended (Table 1) (3–6). The results of these studies revolutionize the field and suggested that “tissue window” might be more personalized than a “time window” to guide precision medicine for ischemic stroke (7, 8). With the rapid development of imaging technology, the ischemic penumbral tissue is now discernible and quantifiable, which provides the possibility to detect salvageable tissue and select the eligible patients for reperfusion therapies (9, 10). A tissue window defined by neuroimaging can serve as surrogate marker for brain physiology in ischemic stroke and facilitate therapeutic decision-making. Here we review the evolution of the time window, address the advantage of tissue window for clinic manage of ischemic stroke, and discuss the roles of neuroimaging in defining a tissue window. We also emphasize metabolic imaging and molecular imaging of brain pathophysiology, and highlight its potential in patient selection and treatment response prediction in ischemic stroke.

**Table 1:** Imaging modalities used in the key clinical trials to expand the therapeutic window of ischemic stroke.

**Study**	**Sample size**	**Time window**	**Imaging modalities**	**Treatment**	**Implications**
EPITHET	101	3–6 h after onset	PWI/ DWI	Alteplase or placebo	Alteplase increased Reperfusion and neurological outcome at 90 days
ECASS III	821	3–4.5 h after onset	CT or MRI	Alteplase or placebo	Alteplase improved neurological outcome at 90 days
MR CLEAN	500	6 h after onset	CTA or MRA	Intraarterial treatment or standard care alone	Intraarterial treatment improved neurological outcome at day 90
EXTEND-IA	70	6 h after onset	CTP	Alteplase with thrombectomy or alteplase alone	Thrombectomy improved reperfusion, early neurologic recovery, and functional outcome
SWIFT PRIME	833	6 h after onset	CTP, or PWI/DWI	tPA, or tPA with endovascular thrombectomy	Thrombectomy showed more effective recanalization than tPA alone
REVASCAT	206	8 h after onset	CT, DWI	Thrombectomy or standard care alone	Thrombectomy reduced the severity of disability
ESCAPE	316	12 h after onset	CT, CTA	Thrombectomy plus standard care or standard care alone	Endovascular thrombectomy benefited the patients with moderate-to-severe ischemic stroke.
DEFUSE-3	182	6–16 h after onset	CTP, PWI/DWI	Thrombectomy plus standard care or standard care alone	Thrombectomy resulted in better functional outcomes than standard medical therapy alone
DOWN	206	6–24 h after onset	DWI, CTP	Thrombectomy plus standard care or standard care alone	Thrombectomy improved the outcomes at 90 days
WAKE UP	503	4.5 h to unknown time of onset	DWI, FLAIR	Alteplase or placebo	Alteplase improved functional outcome at 90 days

*CT, computed tomography; CTP, computed tomography perfusion; CTA, computed tomography angiography; MRI, magnetic resonance imaging; MRA, magnetic resonance angiography; DWI, diffusion-weighted imaging; PWI, perfusion-weighted imaging; FLAIR, fluid-attenuated inversion recovery; tPA, tissue plasminogen activator*.

## The Evolution of Time Window For Reperfusion Therapy

The main aim of existing therapies in ischemic stroke is to restore the blood flow quickly and rescue the potentially salvageable brain tissue. After ischemic stroke, the injured brain is characterized by two major zones: the penumbra and the infarct core. The penumbra is the region around the core that neuronal function is partially preserved (11). The fate of penumbral cells critically relies on regional cerebral blood flow (CBF) and it worsens into infarct core in a time-dependent manner. If reperfusion is established during the early hours, cells in penumbra are salvageable (12). On the contrary, the blood flow in the infarct core declines below to 15–20% of the baseline, this would cause irreversibly damage within the first few minutes of the stroke onset (13). Unfortunately, methods of ischemic core imaging, which is currently in clinical use, are unable to discriminate between incomplete infarction and pan-necrosis. In order to overcome it and revise the clinically relevant parameters more accurately, Goyal et al. suggested replacing the “infarct core” with “ischemic tissue with severity of uncertain viability (SIT-uv)” (14). SIT-uv is considered as tissue that is potentially salvageable by timely reperfusion. Therefore, “time is brain” is still the most important principle guiding reperfusion therapy since the 1990's (15).

Two primary reperfusion strategies have been demonstrated effectiveness: intravenous thrombolysis with tissue plasminogen activator (tPA) and endovascular thrombectomy with stent retrievers (16). However, the benefits of both tPA and endovascular thrombectomy are strongly time-dependent and restricted to only a fraction of stroke patients due to the narrow time window and strict indications (17, 18). In 1995, tPA was originally recommended to treat ischemic stroke within 3 h of the onset (19). Until 2008, Hacke et al. suggested that administration of tPA could be extended to 4.5 h with computed tomographic scan to exclude the patients with hemorrhage or major infarction (20). Because of the narrow time window and strict contraindications, only 2–5% of patients present with ischemic stroke received tPA (21). Recently, guided by perfusion imaging, the window for thrombolysis with alteplase was extended up to 9 h after onset of stroke (EXTEND trial) (22, 23). However, the authors had certain doubts that positive results of the WAKE-up trail nullified the equipoise and terminated the trail early for this reason (3). Furthermore, due to the small number of patients (225 patients from 27 hospitals over 8 years) and because 80% of the patients had large vessel occlusions, the conclusion of EXTEND trail was not reflected in the current guidelines of American Heart Association (AHA). Still AHA recommended taking into account the conclusion of WAKE-UP trail, which stated that patients, who awoke with stroke or had an unclear time of onset which might be more than 4.5 h of the past (>4.5 h from the last known well) could be treated with IV alteplase (24). The eligibility is magnetic resonance imaging (MRI) mismatch between abnormal signal on diffusion-weighted magnetic resonance imaging (DW-MR) and no visible signal change on FLAIR. Present guidelines also endorsed the usage of tenecteplase in patients eligible to mechanical thrombectomy who do not have contraindications for IV fibrinolysis. There is emerging evidence for the non-inferiority of tenecteplase compared to alteplase, although it has not been widely accepted in clinical practice yet (25).

**Figure 1:**
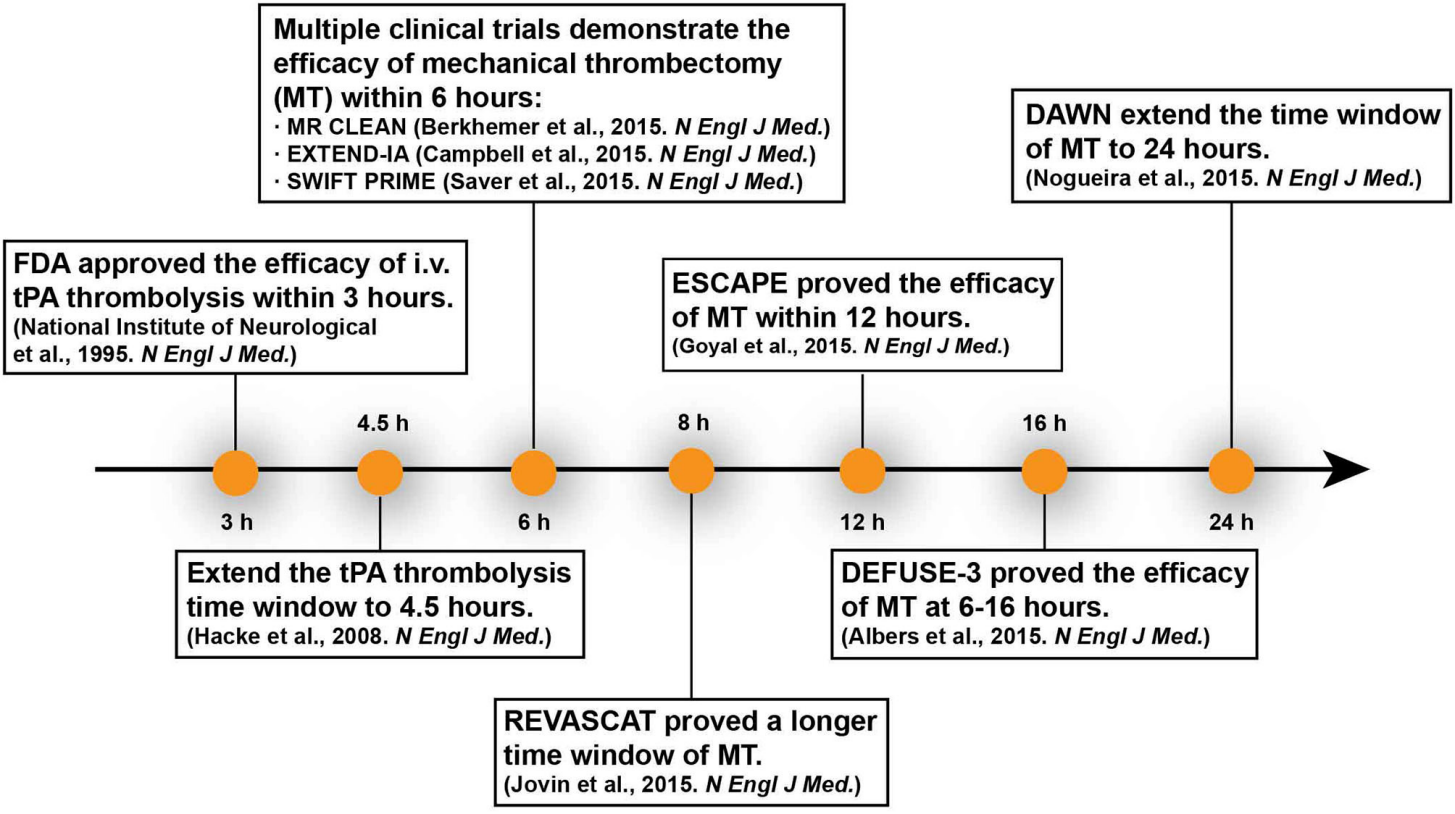
Evolution of the time window for recanalization therapies.

In 2015, it was proven that mechanic thrombectomy with/without intravenous thrombolysis can improve functional outcomes within 6 h after stroke onset (26–28). In patients who have proximal arterial occlusion and small infarct core, mechanical thrombectomy can extend the therapeutic window to 8 h (REVASCAT Trial) even to 12 h (ESCAPE Trial) (29–31). Based on two other recent trials DEFUSE 3 and DAWN, the therapeutic time window can be extended to 24 h since stroke onset (5, 6). Case studies even reported that delayed thrombectomy days or weeks after onset achieved good clinical outcome (32, 33). Recent animal studies proved that delayed recanalization at 3, 7, or 14 days after permanent middle cerebral artery occlusion (MCAO) led to better functional and histological recovery (34). These researches represent a new milestone in acute stroke therapy (Figure 1). Imaging-based patient selection plays a crucial role in the success of these clinical trials and inspires us to rethink the principle “time is brain.”

The salvageability of the affected brain tissue depends primarily on both the duration and the severity of ischemia. The onset-to-treatment time is not the inflexible determinant of reperfusion therapy when takes the collateral flow into accounts. The more extensive the collateral flow, the longer the brain tissue is tolerant to ischemia. However, the collateral flow is highly variable among individuals, which results in variation in tissue susceptibility and therapeutic window. The current “one-size-fit-all” therapeutic time window does not consider the collateral circulation and varied tissue susceptibility, and how to evaluate the viability of the ischemic tissue quickly and accurately is a challenge for the individualized reperfusion therapy.

## Detection of the Penumbra

The penumbra was first experimentally delineated by Astrup in a baboon MCAO model in 1977. Using somatosensory-evoked potentials, he defined the penumbra as an area surrounding the ischemic core in which neurons are affected but have the potential for recovery. Neurons in this area are characterized by low electric activity but sustained energy metabolism, and they do not have noticeable morphological damage (35). Because of the invasive method, it was difficult to translate this experimental concept to clinic and improve stroke diagnosis and treatment. Modern imaging techniques such as positron emission tomography (PET), MRI, and computerized tomography (CT) can distinguish salvageable tissue invasively by measuring hemodynamics and energy metabolism. PET is considered as the “gold standard” for penumbra imaging, however, MRI is more favorable in practice. In addition, CT perfusion is being increasingly used for its low cost and wide availability. The strengths and weaknesses of these imaging modalities on penumbra identification are summarized in Table 2.

**Table 2:** Comparison of strengths and weaknesses of different imaging modalities for penumbra identification.

	**Parameters**	**Strengths**	**Weaknesses**
PET	^15^O-OEF ^15^O-rCBF ^15^O-CMRO_2_	Gold standard	Technical difficulty Invasive procedure Radiation High cost
MRI	DWI/PWI ^23^Na/^1^H MRI Sodium MRI DWI/SWI T2* OC/PWI	Easy-to-use Good spatial resolution High sensitivity Non-invasive No radiation	Time-consuming High cost Inaccuracy
CT	CBF CBV MTT TTP Tmax	Widely accessible Easy-to-use	Radiation Contrast agent required Hard to standardization

*PET, positron emission tomography; MRI, magnetic resonance imaging; CT, computed tomography; OEF, oxygen extraction fraction; rCBF, regional cerebral blood flow; CMRO_2_, cerebral metabolic rate of oxygen; DWI, diffusion weighted imaging; PWI, perfusion weighted imaging; SWI, susceptibility-weighted imaging; OC, oxygen challenge; CBF, cerebral blood flow; CBV, cerebral blood volume; MTT, mean transit time; TTP, time to peak; Tmax, time to maximum*.

### PET

The existence of penumbra in stroke patients was demonstrated for the first time by PET. In 1981, Baron et al. observed decreased CBF and increased oxygen extraction fraction (OEF) in an ischemic stroke patient by PET and coined this modality as “misery perfusion” which indicated potential viable tissue (36). This area with increased OEF was the original definition of penumbra. Labeling arterial blood sample with ^15^O allows PET to assess the regional CBF, OEF and cerebral metabolic rate of oxygen (CMRO_2_) (CMRO_2_ = CBF × OEF × arterial oxygen content) and determine the penumbra. Early PET studies classified the ischemic tissue into 3 regions depending on CBF rate: the infarct core with CBF <12 ml/100 g·min, the penumbra with CBF of 12–22 ml/100 g·min and the oligemia with CBF >22 ml/100 g·min (37–40). In practical applications, the extent of penumbra is dynamic and time dependent process, which varies with the severity and duration of ischemia. CBF value only reflects the reperfusion status at the time of imaging. An initial severe ischemia may show a normal appearing CBF value because of partial restoration of blood flow, but the ischemic tissue has already irreversibly damaged (37, 41). The advanced ^15^O-O_2_ PET can detect OEF and distinguish viable tissue from core infarction. Thus, the mismatch between CBF and oxygen metabolism is usually considered as the *in vivo* hallmark of penumbra region, which maintains transient oxygen supply while suffering severe hypoperfusion (40, 42). Recently, some advanced approaches have been used to identify the penumbra. ^11^C-flumazenil (^11^C FMZ), a marker of cortical neuron integrity, combine with ^15^O-H_2_O PET can detect early neuronal death irrespective of time elapse and without arterial blood sampling. Based on the specific metabolic parameters, it is well-accepted that PET is the gold standard for determining the penumbra (43, 44).

However, detection of penumbra by PET has several limitations, such as technical difficulty, invasive procedures, exposure to radioactivity and high cost, which prevent PET from broad acceptance in clinical routine (44). Therefore, both improvement of current methods and the development of other imaging modalities are needed.

### MRI

**Figure 2:**
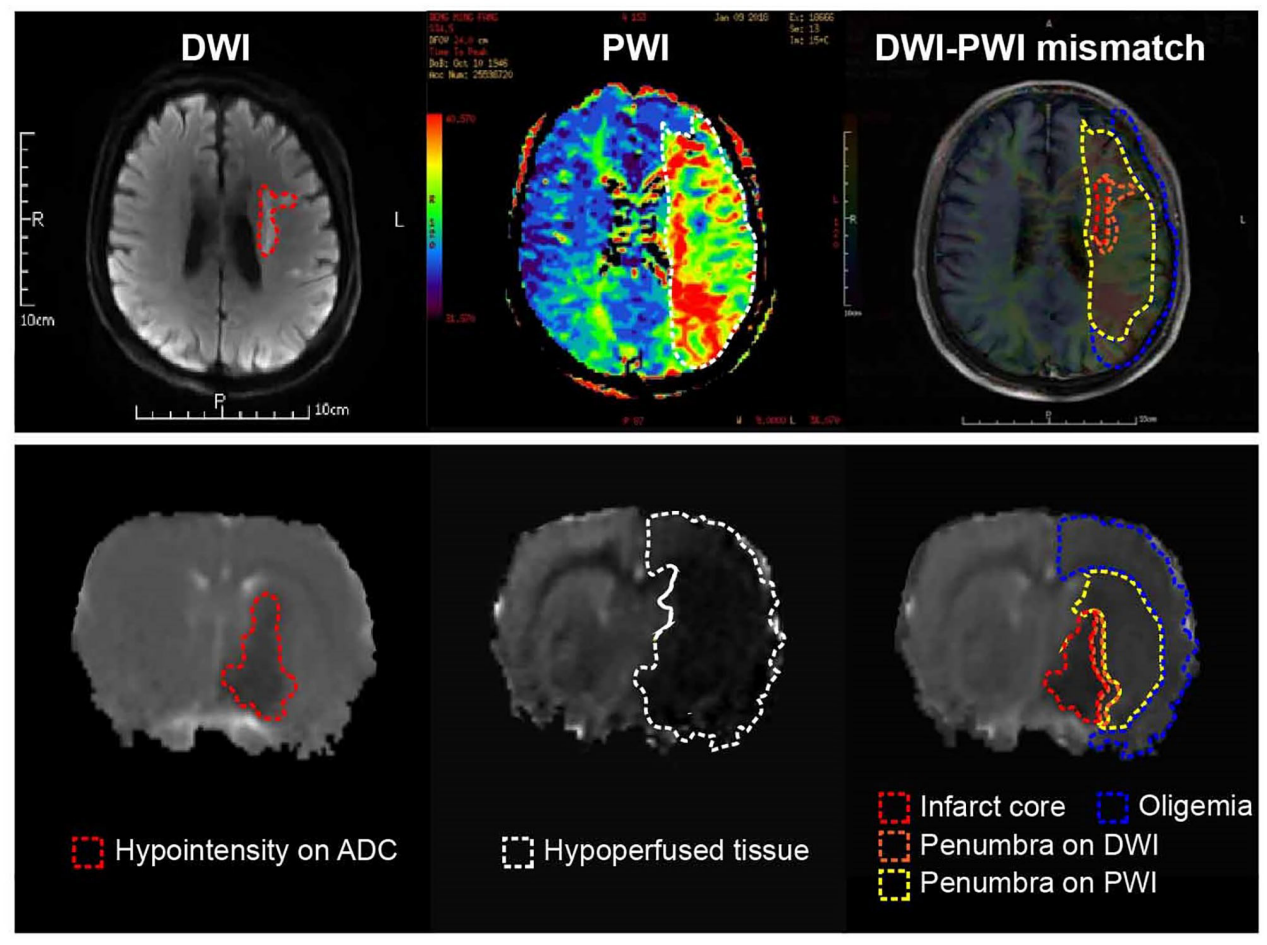
Current concept of the ischemic penumbra. Both clinical (Upper) and experimental (Below) MRI data showed early abnormality on DWI equals the infarct core plus a part of tissue at risk (penumbra), and the perfusion deficiency on PWI includes part of the region of benign oligemia. MRI, magnetic resonance imaging; PWI, perfusion-weighted imaging.

**Figure 3:**
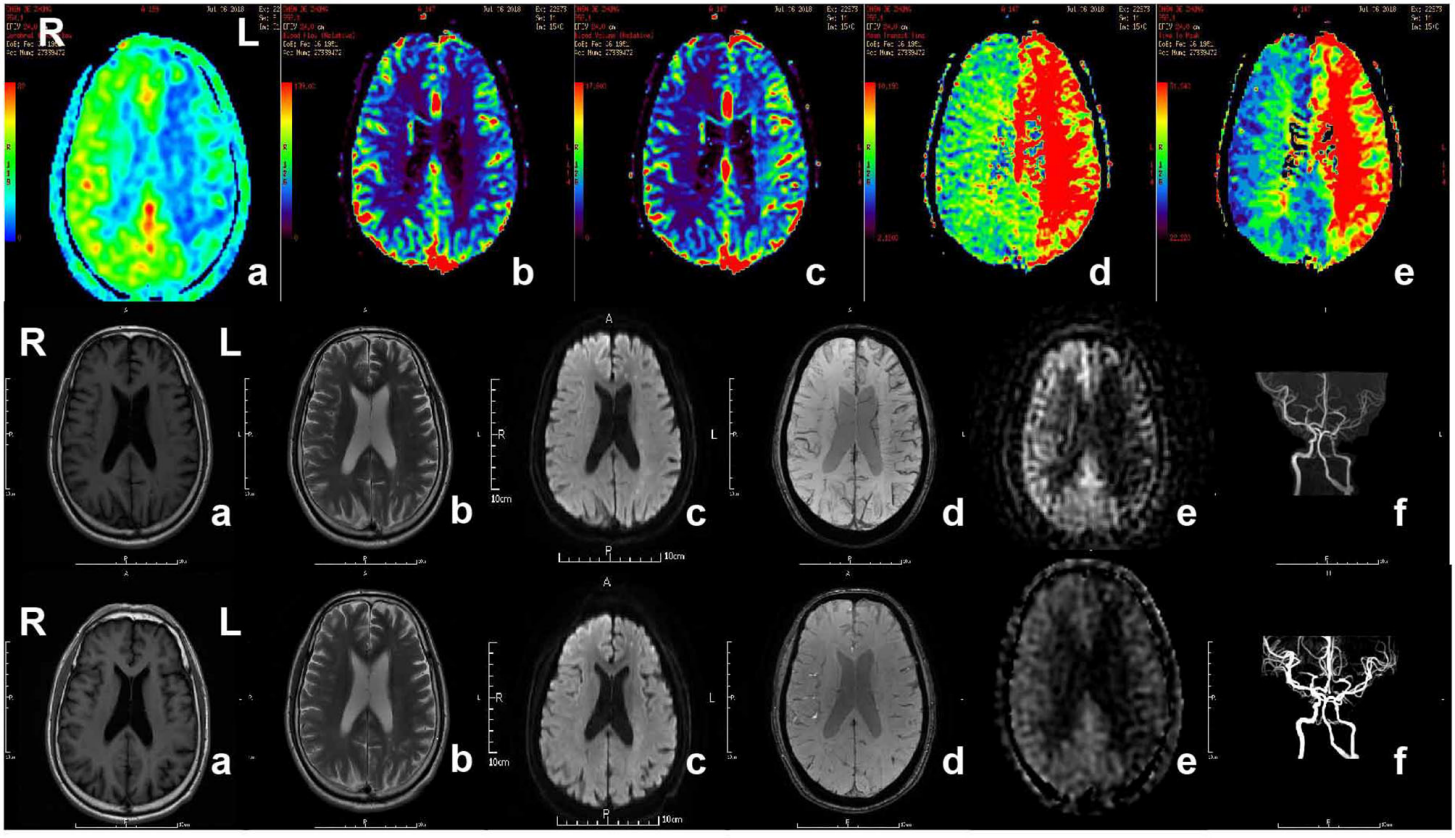
MR imaging of the patient with acute ischemic stroke. The patient, 71-year-old male, suffered from weakness of right limbs, alalia and was unable to walk 3 h prior to the imaging. Medical history included hypertension, diabetes and varicosity of both lower extremities. Physical examination showed right facial paralysis, the muscle strength of right limbs was grade I, right Babinski sign (+), Chaddock sigh (+), and NIHSS score was 13. The diagnosis of the patient is acute ischemic stroke. The patient was given multimodal MRI and MRA before and after recanalization. The first row showed perfusion-weighted imaging. a and b, Lower CBF in left cerebral hemisphere. c, Higher CBV in left cerebral hemisphere. d, Longer MTT in left cerebral hemisphere. e, Longer TTP in left cerebral hemisphere. The second row and third row showed MRI and MRA before recanalization and after recanalization, respectively. a, b, and c, In the left frontal lobe and lateral ventricle had sporadic dots with slightly longer T1 signal, longer T2 signal higher signal, respectively, in T1WI, T2WI, DWI. d, No cerebral microbleeds in SWI. e, Original ASL. f, MRA showed the intracranial segment of the left internal carotid artery and the left middle cerebral artery were significantly narrow in the second row, while recanalization got in left internal carotid artery and middle cerebral artery. MR, magnetic resonance; MRA, magnetic resonance angiography; SWI, susceptibility weighted imaging; ASL, arterial spin labeling; CBV, cerebral blood volume; CBF, cerebral blood flow; TTP, time to peak; MTT, mean transit time.

Compare to PET, MRI has better spatial and temporal resolution, and no risk of radiation exposure of patients. MRI has largely replaced PET in acute stroke imaging in clinic (45). The MRI-based perfusion-diffusion mismatch (PDM) is a surrogate of PET-based penumbra evaluation (Figure 2). Diffusion weighted imaging (DWI) refers to the visualization of random Brownian movement of water molecules in brain tissue. Lower diffusion coefficients generally resulted from energy failure and subsequent cytotoxic edema, and it is suggested to delineate infarct core tissue that irreversibly damaged (Figure 3) (46). Perfusion weighted imaging (PWI) measures brain perfusion dynamically with such parameters as CBF, cerebral blood volume (CBV), mean transit time (MTT) and time to peak (TTP) (Figure 3) (47). PWI abnormality provides the information on both infarct core and the surrounding hypoperfused tissue. Volumetric-based PDM is usually defined as a mismatch ratio of PWI/DWI ≥1.2, which is postulated to represent the penumbra that locates outside the infarct core but is at risk of infarction (48). The concept of PDM has been proved practically in both experimental and clinical studies (49–51). Early clinic studies have shown that salvage of brain tissue delineated by the PDM improved neurological functions (52, 53), and selection of patients with PDM increased the rate of reperfusion and achieved favorable clinical response when treated within 6 h (54, 55). However, this surrogate marker of penumbra is challenged by several limitations (44). Comparative PET/MRI studies confirmed the mismatch area imprecisely depicts elevated OEF and overestimated the penumbra from benign oligemia defined by PET. Because of the wide variation in thresholds, DWI also overestimates the infarct core by including part of the penumbra (56–58). And as the viability and metabolic state of brain tissue strongly depends on the duration of ischemia, for those “wake-up” patients who didn't know stroke onset time (SOT), it is hard to set threshold of the parameters [such as TTP and time to maximum (Tmax)] (57).

Accumulating evidence has shown that there was no clear association between PDM and penumbra (59–62), and the inaccuracy of PDM in defining penumbra may be responsible for the failure of some reperfusion and neuroprotection therapies in clinic (63, 64). It is urgent to develop novel imaging paradigms that can serve as a clinical marker of penumbra. Several attempts have been made to improve the accuracy of PDM in penumbra predicting. Combined ^23^Na-MRI to ^1^H-MRI was developed to complement PDM and serve as a viability marker for penumbra detection in several animal models. Tissue sodium concentration increased in the core and decreased in the penumbra so that the viable penumbra could be differentiated from the core in transient MCAO rats (65, 66). In addition, it has been proposed that sodium MRI may help determine the SOT by calculating this retrospectively (67, 68). Susceptibility-weighted imaging (SWI) is also used to identify the penumbra in stroke patients. SWI detects the paramagnetic susceptibility difference between deoxygenated and oxygenated hemoglobin, which reflects the OEF of brain tissue (69). DWI-SWI mismatch is shown to be a promising marker for evaluating penumbra (70). In addition, by mapping the ratio changes of deoxyhemoglobin/oxyhemoglobin, ${\text{T}}_{2}^{*}$ oxygen challenge combined PWI assessed the viability of penumbra serially and showed advantages over PDM for penumbra detection (71).

### Computed Tomography Perfusion (CTP)

**Figure 4:**
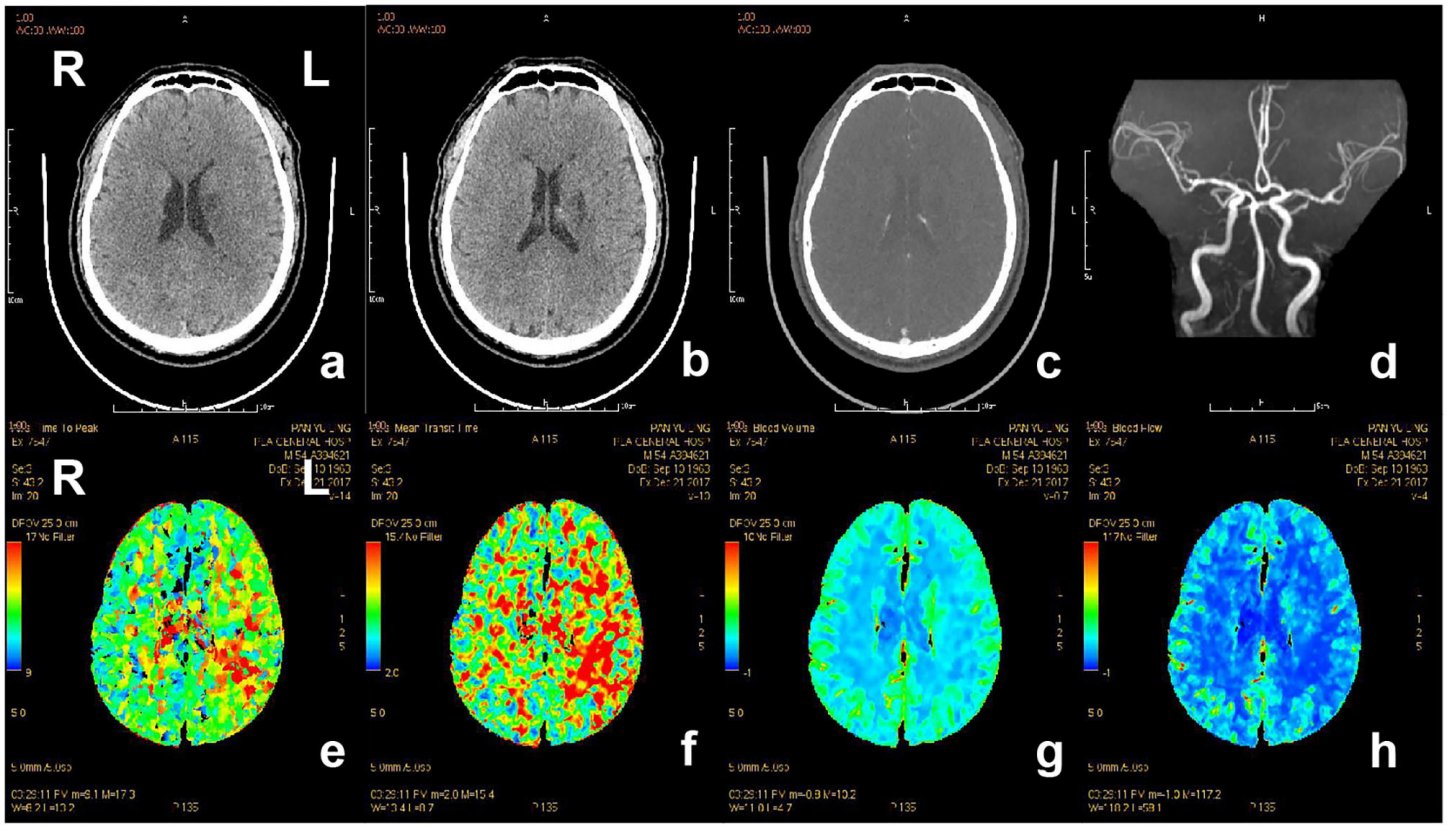
CT and CTP imaging of the patient with acute ischemic stroke. The patient, 80-year-old-female, suffered from weakness of right limbs, alalia and was unable to walk 3.5 h prior to the imaging. Medical history included hypertension, atrial fibrillation and gout. Physical examination showed right facial paralysis, the muscle strength of right limbs was grade I, right Babinski sign (+), and NIHSS score was 10. The diagnosis of the patient is acute ischemic stroke. The patient was given multimodal CT and MRA before recanalization. a and b, The left bilateral basal ganglia and bilateral frontal lobe showed sporadic dots and patches with low-density shadow on non-contrast CT scan. c, Contrast-enhanced CT showed no abnormal enhanced shadow. d, MRA showed the lumen of bilateral middle cerebral artery was not smooth, the left cerebral artery M1 segment had multiple stenosis, and distal branch vessels were disappeared. e and f, Prolonged TTP and MTT in the area of left middle cerebral artery. g and h, CBF and CBV showed no significant abnormal area. CT, computed tomography; CTP, computed tomography perfusion; CBV, cerebral blood volume; CBF, cerebral blood flow; TTP, time to peak; MTT, mean transit time.

CTP is an imaging technique that is increasingly used for determination of infarct core and penumbra in acute ischemic stroke patients. The clear advantages of this technique are its easily accessibility and fast acquisition (72). Similar to PWI MRI, raw data of CTP is also displayed in parameter maps, including CBF, CBV, MMT, TTP, and Tmax. Regions with dramatically reduced CBF or CBV correspond to the core infarction, while regions with prolonged MTT, TTP, or Tmax delineate the penumbra (Figure 4) (73). However, there is significant variability in CTP technique between different CT scanners, processing software and prior institutional optimization, and this results in controversy about the accurate measurement of penumbra (74). Moreover, due to the delay of the arrival of contrast to brain, CBV calculation always results in an overestimation of the core lesion that leads to an underestimation of penumbra (75). CTP still has the risk of radiation exposure and toxicity of the contrast agent.

## Metabolic Imaging of Ischmeic Stroke

For patients excluded from reperfusion therapy due to exit of the therapeutic time window established by imaging strategy mentioned above, there is another opportunity to expand the treatable population: selecting of the patients by advanced physiologic imaging. Both MRI-based PDM and CTP depend on selecting threshold values of blood flow to differentiate the penumbra from infarct core and benign oligemic brain tissue. And these thresholds change with the evolution of stroke. So far, there are no validated thresholds that accepted for routine penumbra imaging in the clinical setting. Parameters which display the physiology of brain immediately and independently of the onset-time are in urgent need to delineate penumbra accurately and guide the precision therapy in stroke. When the blood flow is compromised, energy metabolism disturbance occurs within seconds as brain has very limited supply of energy producing substances and relies on oxidative metabolism to meet its tremendous energy requirements (76, 77). The metabolic stress induces ionic perturbations and oxidative stress which trigger the cascade of pathophysiological events ultimately resulting in neuronal death (78). Accumulating evidence has suggested that energy status is associated with cell survival and determines the fate of ischemic tissue (79–81). Direct measurement of the metabolic status provides more accurate information to delineate the viable tissue. Therefore, energy metabolism can serve as a direct indicator of the salvageable tissue in the penumbra zone. Quantification of cerebral oxygen metabolism has shown great promise in revealing the viability of ischemic tissue during stroke. Several imaging modalities, such as PET and MRI, have been applied to image cerebral oxygen metabolism in both experimental research as well as clinical practice (82). These imaging methods and parameters they can detect are summarized in Table 3. Furthermore, we review the most recently advances in metabolic imaging, which may greatly facilitate routine clinical applications to guide optimal therapy decision for acute ischemic stroke as well as subacute or chronic stroke with permanent large vessel occlusion.

**Table 3:** The three major imaging methods for acute stroke and physiological parameters they can detect.

	**Parameters**	**PET**	**MRI**	**CT**
Metabolism	Oxygen metabolism	^15^O-OEF ^15^O-CMRO_2_	OCI	
	Neuronal death	^11^C-FMZ		
	Hypoxia	^18^F-MISO Cu-ATSM		
	pH		APT-MRI ^13^P MRS	
	Lactate		^1^H MRS ^13^C MRS	
	NAA		^1^H MRS	
	PCr/Pi		^13^P MRS	
	ATP		^13^P MRS	
Pathophysiology	Excitotoxicity		^1^H MRS GluCEST	
	Apoptosis		^1^H MRS	
	Inflammation	Cu-ATSM TSPO	SPIO/USPIO MNP-PBP MPIO	
	BBB leakage		DCE-MRI	DCE-CT Micro-CT

*FMZ, flumazenil; MISO, misonidazole; Cu-ATSM, Copper-based Radiopharmaceuticals; APT, amide proton transfer; MRS, MR spectroscopy; NAA, N-acetylaspartate;PCr, phosphocreatine; Pi, inorganic phosphate; ATP, adenosine triphosphate; CEST, chemical exchange saturation transfer;S PIO/USPIO, small and ultrasmall superparamagnetic iron oxide particles; MNP-PBP, magnetic nanoparticle-P-selectin binding peptide; MPIO, microparticles of iron-oxide; TSPO, translocator protein; DCE, dynamic contrast-enhanced*.

### PET Metabolic Imaging

As mentioned above, ^15^O multi-tracer PET can provide the tomographic distributed imaging of brain oxygen extraction and metabolism. More importantly, it is the reference standard for quantitative evaluations of OEF, CMRO_2_, CBV, and CBF. In the late 1970's, scientists successfully measured the regional brain CBV and CBF, and oxygen metabolism in stroke patients with ^15^O-labeled PET, and distinguished the severely hypoperfused but potentially salvageable tissue from the irreversibly damaged (83–85). Using ^15^O-H_2_O PET, Heiss et al. found that the misery perfused tissue was salvaged by early intravenous thrombolysis (86). This study is a millstone in stroke research. It revolutionized the management of acute stroke patients by demonstrating the positive result of rt-PA. However, due to the technical complexity, ^15^O PET is not widely performed in clinical settings, with issues involving requirement of on-site cyclotron and radiochemistry facility because of the short half-life of ^15^O (2 min), real-time artery blood sampling and analyzing to obtain the regional CBF (rCBF), as well as complex post processing (87). Efforts have been made to streamline the ^15^O-PET examination for routine clinical practice, including: (i) quantitative voxel-by-voxel maps of rCBF without a direct arterial input function (88); (ii) shortening the clinical examination period by dual-tracer (${\text{H}}_{2}^{15}$O and ^15^O_2_) autoradiography approach (89); and (iii) developing non-invasive techniques to assess CBF, OEF, and CMRO_2_ (90). Attempts were also made in animal stroke studies. Methodological inventions, such as intravenous administration of injectable ^15^O_2_ and inhalation of ^15^O_2_ gas, had been tried to facilitate the evaluation of CMRO_2_ and OEF in small animals (91–93). These developments potentiate the possibility of using PET in clinical routine to expand the treatable stroke patients in early stage.

The improvement of the radiotracers has revolutionized the use of PET in measuring OEF and CMRO_2_ and mapping the cerebrovascular reserve and the penumbra. The newly developed PET ligands, including radio-labeled FMZ, radio-labeled fluoromisonidazole (FMISO), and copper-based radiopharmaceuticals (Cu-ATSM) have been explored to delineate the disease in preclinical and clinical research. In 1997, ^11^C-FMZ PET was used to indicate the development of infarction in cat stroke model and showed the potential to select eligible patients for early therapeutic intervention (94). In 2000, a clinical trial proved that 11C-FMZ PET was able to differentiate the viable tissue from the irreversibly damaged at the early stage of acute stroke (95). In this study, the areas with reduced perfusion but preserved ^11^C-FMZ binding could benefit from reperfusion therapy, while the areas with ^11^C-FMZ uptake defects were permanent lesions. Thiel et al. reported that ^11^C-FMZ PET could be used to estimate rCBF in ischemia without arterial input function (96). However, the application of ^11^C-FMZ PET in clinical practice is limited by several issues: the requirement of cyclotron to produce ^11^C, the regional expression of benzodiazepine receptors in cerebral cortex, and the low affinity of ^11^C-FMZ to bind with its receptor at the acute phase of ischemia/reperfusion (97). New PET tracers are still in urgent need to meet the clinical requirements.

Compared with^11^C-FMZ PET, ^18^F-FMISO PET is more broadly used (98). In a preclinical study, ^18^F-FMISO microPET was used to map the brain hypoxia in the acute stage of permanent distal MCAO rats, and supported that ^18^F-FMISO might be a marker of core area as well as of penumbra (99). Besides, ^18^F-FMISO uptake was also used to predict the tissue fate. The patients without ^18^F-FMISO uptake had no infarct growth on the follow-up DWI, while those with abnormally increased ^18^F-FMISO uptake showed grown infarct (100). And white matter was reported to take up more ^18^F-FMISO than gray matter, indicating stronger resistance to ischemia than gray matter (101). However, the penumbra outlined with ^18^F-FMISO may be overestimated. When using ^18^F-FMISO in ischemic stroke patients, large regions of hypoxic tissue was found surrounding the ischemic core, which spontaneously reverted back to normal (102). The major drawback of ^18^F-FMISO PET is the slow kinetics of ^18^F-FMISO, which requires 2–3 h to clear it from the hypoxic tissue. A faster kinetic and metabolic rate tracer is needed for metabolic imaging. Compared with nitroimidazole, Cu-ATSM is rapidly washed out, and the imaging can be finished with 20–30 min after injection (103). Cu-ATSM has also been proved to modulate inflammation and has therapeutic potential in experimental stroke (104).

### MRI Metabolic Imaging

Studies have investigated MR-based PWI and DWI, and suggested the PWI-DWI mismatch regions as potentially salvageable tissue (51). This clinical routine protocol does not define metabolic activity directly; however, PWI and DWI clearly indicate different metabolic regions. The following sections will carefully discuss related major MRI metabolic imaging techniques, which have been summarized in Table 2.

### Magnetic Resonance Spectroscopy

MRS acquires the signal arising from brain metabolites by analyzing molecules such as hydrogen ions or protons. Because of high spatial and temporal resolution, proton MRS (^1^H MRS) is the more commonly used. The most assessed metabolites with potential value for clinical stroke evaluation are lactate (1.30 ppm) and tNAA (2.02 ppm) (105–107). Preclinical studies have shown that levels of total N-acetylaspartate (tNAA) decrease to 50% in the first 6 h after ischemic stroke, followed by a milder decrease to 20% for the subsequent 24 h, and gradually returned to 30% until 7 day (107). Clinically, the concentration of tNAA in penumbra and in core infarction may even decrease below the level of detection (108, 109). Severely decreased tNAA is related to serious clinical syndrome and extensive infarction, which means poor clinical outcome (110). Lactate is the end product of anaerobic glycolysis and rises within minutes after ischemic stroke. Elevated lactate in the core of ischemic tissue is positively related to the final infarct size and neurological deficits (111). The increase of lactate accompanied with reduction of tNAA was observed in patients with large infarction and poor outcome (110). Therefore, the level of lactate and tNAA is important for evaluating the severity of stroke and predicting the recurrence of ischemic events (112). Interestingly, a recent MRS stroke animal study suggested that ML3 (bis-alyllic protons of polyunsaturated fatty acids, 2.80 ppm), which was detected of a significant increase at 7 days after stroke, may be a non-invasive surrogate biomarker of cumulative apoptosis in stroke, which could be used as a clinical predictive marker (107).

^13^P MRS is also used to evaluate brain energy metabolism in ischemic stroke by assessing the high energy phosphate metabolism, particularly adenosine triphosphate (ATP) and creatine phosphate (PCr) (113). A gradual decrease in ATP was only exhibited in severe stroke, not mild stroke (114). The ratio of PCr to inorganic phosphate (Pi) (PCr/Pi) showed a precipitous decrease during ischemia as well as reperfusion (114). Cerebral intracellular pH can also be measured by ^13^P MRS. It was calculated by the chemical shift (δ) of the Pi resonance peak relative to the PCr resonance peak (115).

Though MRS is of great value in assessing the severity of ischemia and predicting the risk for recurrence, low signal-noise ratio, long sequence duration and the risk of lipid contamination make MRS not suitable for routine assessment of acute ischemic stroke patients.

### Oxygen Challenge Imaging

Oxygen challenge imaging (OCI) is based on blood oxygen level-dependent (BOLD) contrast MRI that reflects the changes in blood oxygen saturation. OCI uses transient hyperoxia during ${\text{T}}_{2}^{*}$-weighted MRI to present dynamic changes in deoxyhemoglobin concentration. Therefore, tissues in the penumbra exhibit an increase in ${\text{T}}_{2}^{*}$ signal intensity, with diminished or absent ${\text{T}}_{2}^{*}$ signal intensity in the infarct core. Time to peak value from OCI offers additional information to facilitate the identification of at-risk tissue in ischemic stroke rats (116). The region of OCI response was reported to be larger than the PWI-DWI mismatch region (117). The ${\text{T}}_{2}^{*}$ OCI was developed using 100% normobaric hyperoxia and has showed clinical translational potential for stroke diagnosis. However, inhaled 100% oxygen induces sinus artifacts in the front lobe. Decreasing the concentration of inhaled oxygen can decrease these artifacts, but makes more difficult to distinguish the penumbra from surrounding tissues (117). Recently, the combination of 40% oxygen and perfluorocarbons and fluorinated hydrocarbons with respiratory gas significantly enhanced ${\text{T}}_{2}^{*}$ response to 40% oxygen in ${\text{T}}_{2}^{*}$ defined penumbra (118). The same group also developed the GOLD (Glasgow Oxygen Level Dependent) diagnostic imaging method by using ${\text{T}}_{2}^{*}$oxygen challenge (${\text{T}}_{2}^{*}$OC, 100% inhaled oxygen) combined with lactate change MRS technique (119). This method worked concurrently to identify the salvageable tissue in penumbra based on the glucose metabolic status in MCAO rats. However, the disadvantages of OCI are, as mentioned above, the poor signal-to-noise due to the limited oxygen that delivered to the tissues and the artifacts caused by the paramagnetic effect of 100% O_2_ within the paranasal sinuses (120).

### pH-Weighted Imaging

Zhou et al. developed a new MRI approach which was predominantly sensitive to the intracellular pH changes (121). This method benefited from the chemical exchange processes, which amide protons transfer between cellular peptides and proteins in a pH-dependent manner. Because amide proton transfer (APT) is pH dependent, measures of APT may be used to measure pH value (122). Acute ischemic stroke causes an accumulation of lactic acid and results in the decrease of pH, which could be the earliest sign for the tissue at risk. Accordingly, there were research data which suggested that pH imaging could be used to define the ischemic penumbra. The hypoperfused tissue with normal ADC and low pH may represent ischemic penumbra (121, 123, 124). A further study suggested that an additional pH-weighted imaging with PWI-DWI was superior to PWI-DWI alone to predict the tissue outcome in ischemic stroke rats (125).

Although most early studies, which investigated pH-weighted imaging technique, were performed in rodents, there is increasing translation of this technique to human studies. Tietze et al., for the first time, demonstrated that clinical application of pH-weighted imaging in acute stroke patients was possible and could be quantified, which carried potential for providing additional information on metabolic changes in acute ischemia (126). Subsequently, scientists from Oxford successfully identified the ischemic penumbra using pH-weighted magnetic resonance imaging (127).

Although studies related to pH-weighted imaging have provided important insights in the pathology of acute stroke, they currently cannot be applied in clinical routine due to their technical limitations, such as hardware constraints of human MRI scanner (short repetition time and strong radio-frequency saturation power), acquisition protocols selection (single-slice or volumetric APT imaging) and analyzing techniques (128). Consequently, pH-weighted imaging studies have paid more attention to develop better quantifying approaches and improve the APT MRI sensitivity to pH, thus, the acidosis in ischemic penumbra can be more reliably delineated (128–130).

### Other Mmetabolic Imaging Techniques

There are many other imaging modalities that are at early stage of ischemic metabolic imaging, such as sodium imaging, PET ^17^O imaging, and MR-derived cerebral metabolic oxygen index (MR COMI) (131). While metabolic imaging is promising, emerging imaging technologies require considerable validation to consider how they fit into the current imaging protocols and what information they accurately provide to guide the recanalization therapy. Many of these techniques will require technical refinement before they can be used in clinical acute ischemic stroke.

## Molecular Imaging of Pathophysiology

Ischemia causes the shortage of glucose and oxygen and subsequently depletion of ATP, which result in the dysfunction of sodium-potassium pump and membrane depolarization (132). That induces multiple pathophysiological cascades, including excitotoxicity, apoptosis, acidosis, blood-brain barrier (BBB) leakage, and immune response, which lead to ischemic neuronal loss (133).

Several imaging techniques have been used in the visualization of ischemic stroke pathophysiology (134). For example, ischemia results in marked reduction of tissue pH that triggers neuronal death (135). As mentioned above, pH-weighted MRI can detect the changes of tissue pH value that reflect the progress of acidotoxicity. MRS can evaluate tissue levels of lactate and ML3 that on this way estimate the status of acidosis and apoptosis. Current developments of pathophysiology imaging facilitate the *in vivo* assessment of pathophysiological markers and therapeutic targets after stroke, and provide the opportunities for the translation of multimodal imaging strategies in stroke diagnosis and treatment. The pathophysiological parameters that can be detected are summarized in Table 2.

### Visualization of Excitotoxicity

Excitotoxicity caused by excessive release of glutamate is one of the major culprits that responsible for the neuronal death and neurological deficits after stroke. The levels of glutamate can be detected by ^1^H MRS. In MCAO model in rats, Ramos-Cabrer et al. demonstrated that the levels of glutamate increase in center of the infarction core and then spread to the peri-infarction. Within 24 h after stroke, glutamate levels decreased significantly in the infarct core area, whereas regular levels were detected in the periphery of the core lesion (136). A clinical research found the differences of glutamate levels between infarct core and reperfused ischemic penumbra. In the ischemic stroke patients who received intravenous tPA within 4.5 h, high glutamate concentrations in peri-infarct were observed in the hyperperfused patients, while glutamate concentrations were low in the non-hyperperfused patients (137). The evaluation of glutamate depends on the magnetic field strengths. At low magnetic field scanner, the peak of glutamate and glutamine are consecutive that cannot distinguish glutamate from glutamine. At field strengths of 3.0 T or higher, the separation of glutamate and glutamine is feasible. Besides, ^1^H MRS technique requires long acquisition times and has low spatial resolution.

Recently, a new MRI technique for imaging glutamate has been developed based on chemical exchange saturation transfer (CEST) effect. The CEST effects of amide and hydroxyl protons have also been used to measure pH value changes after ischemic stroke. It has been demonstrated that middle cerebral artery occlusion (MCAO) induced about 100% elevation of glutamate CEST (GluCEST) in the ischemic tissue compared with the contralateral side in rats. This method images the relative changes of glutamate and has the advantages of high spatial and temporal resolution. However, GluCEST imaging is only achievable in the human brain in ultrahigh field (7.0 T) and is not currently accessible in the clinic (138).

### Monitoring the Neuroinflammation and Immune System

The immune system plays a pivotal role in the response to ischemia and the eventual recovery of function (139). The complex cascade of immune cells and inflammatory factors contribute to the breakdown of BBB. After stroke, microglia immediately respond to the ischemic insult, followed by the proliferation of macrophages, dendritic cells, and lymphocytes. With the occurrence of BBB breakdown, neutrophilic cells permeate the infarct and peri-infarct region. The immune cells release excessive pro-inflammatory cytokines (i.e., TNF-α and IL-1β) and produce large amounts of free radicals, which contribute to the upregulation of cell adhesion molecule and further propagate the inflammatory response (133). Additionally, inflammation elevates production of matrix metalloproteins (MMPs) and myeloperoxidase, both of which are major factors leading to BBB breakdown.

After ischemic stroke, the spatiotemporal profile of neuroinflammation with cellular and molecular MRI has been increasingly explored. In cellular and molecular MRI, paramagnetic contrast agents such as gadolinium chelates and small particles of iron oxide were used to detect specific leukocyte populations or molecular inflammatory markers after stroke. Several pre-clinical and clinical studies have demonstrated the application of contrast agents to image the monocyte infiltration after ischemic stroke. The most common strategy for labeling circulating monocytes is the administration of iron oxide nanoparticles. For the labeling, two kinds of particles can be used: small and ultrasmall superparamagnetic iron oxide particles (SPIO and USPIO, respectively) (140–144). SPIO and USPIO shorten the transverse relaxation times T_2_ and ${\text{T}}_{2}^{*}$, and the cells taken up SPIO or USPIO present hypointense on T_2_ or ${\text{T}}_{2}^{*}$-weighted images. Rausch et al. found that USPIOs distributed in patches within the lesion and surrounding area on the first 2 days. On day 4, USPIOs expanded within the lesion core. On day 7 they were found predominantly within the boundary area. This strategy has been used in some human studies to detect macrophage activity in stroke patients (140, 141). With the target-specific contrast agents, molecular MRI have shown the potential to examine inflammation markers *in vivo* in experimental stroke. Jin et al. designed a magnetic nanoparticle-P-selectin binding peptide (MNP-PBP) to image endothelial P-selectin and E-selectin. MNP-PBP showed a notably greater T_2_ effect in the infarction and had a tighter binding affinity with selectin (145, 146). Vascular cell adhesion molecule (VCAM-1) and MMPs are other targets for neuroinflammation imaging. In a pre-clinical study, researchers developed microparticles of iron-oxide (MPIO), an MRI contrast agent that could bind with VCAM-1 on the cerebral vascular endothelium and visualize the expression of VCAM-1 (147). The result showed the spatial extent of VCAM-1 was considerably larger than the lesion core area measured by DWI MRI. The authors thought that this molecular MRI imaging of VCAM-1 might include both ischemic core and potentially salvageable penumbral regions.

Microglial activation can be monitored by PET imaging with related molecular biomarkers. The 18 kDa translocator protein (TSPO) system is the most commonly used target system for neuroinflammatory imaging. Due to the activation of microglia, the TSPO density is elevated after ischemic stroke. Furthermore, it has been demonstrated that using [^11^C] vinpocetine, a prospective radio ligand of TSPO, the regional changes of TSPO can be measured in the brain of ischemic stroke patients (148). The elevated level of TSPO which indicated the activated microglia was found both in the ischemic core and peri-infarct area.

As far as imaging modalities concern there are only a few options, which allow do discriminate different cell types in the infarcted tissue of patients. Molecular MRI has shown promising futures regarding monitoring of inflammatory cells such as neutrophils, leukocytes and microglia, but it is unable to provide the information in regards of cell viability. Futhermore, the molecular MRI is currently not highly specific and the interpretation of obtained results is sometimes challenging. The toxicity of contrast agents is another concern. All these shortcomings complicate the clinical implementation of this imaging technique.

### Imaging Blood-Brain Barrier Leakage

Restoration of blood flow can induce reperfusion injury, reperfusion injury is one of the events that compromises the BBB. The leakage of BBB, which has been reported have a biphasic pattern, can cause severe brain edema and hemorrhagic transformation (149, 150). MRI and CT are the most widely used clinical imaging tools to evaluate BBB disruption by detecting the extravasation of the intravenously administered small molecular weight contrast agents (151).

Dynamic contrast-enhanced MRI (DCE-MRI) is considered as the gold standard MRI approach to evaluate BBB permeability (152). Gadolinium-diethylenetriamine penta-acetic acid and its variant gadolinium-diethylenetriamine penta-acetic acid-bis (methylamide) are the most used contrast agents that are given as intravenous bolus injection. The accumulation of contrast agents in the extracellular matrix of ischemic tissues results in increased longitudinal relaxation rate and hyperintensity in T_1_-weighted MRI. DCE-MRI exploits this T_1_ enhancement to extract quantitative or semi-quantitative information regarding BBB integrity (153). Several pre-clinical studies using DCE-MRI assessed BBB integrity after ischemic stroke. The results consistently have shown a biphasic pattern of BBB permeability after ischemic stroke. Compared to sham-operated animals, the BBB permeability on ipsilateral striatum increased at 4 h after the onset of ischemic stroke. Compared to the 4 h value, a significant decline of stroke-induced BBB disruption was observed at 24 h. Another rise of BBB permeability followed at 48 h. At this time point, the BBB disruption was more prominent than at both 4 and at 24 h after stroke induction (154). The mechanisms of this partial recovery in BBB function are not completely understood. Besides, biphasic pattern of BBB permeability has not been categorically confirmed in stroke patients.

CT can also be used to evaluate BBB integrity. Similar with DCE-MRI, DCE-CT involves intravenous injection of an iodinated contrast agent and voxel-wise measurement of attenuation coefficient as a function of time (155). In the clinical setting, the accessibility and the fast scanning speed of CT makes it the first choice for making treatment decisions for ischemic stroke. BBB disruption can potentially be assessed by incorporating a DCE-CT protocol into the initial CT imaging of a patient. Recently, Park et al. developed a new *in vivo* micro-CT which uses iopromide to visualize the leakage of BBB. The new micro-CT BBB imaging technique has a high resolution and sensitivity (156).

Although some other imaging techniques (such as PET and optical imaging) have been also used to evaluate BBB permeability, their limitations, such as low resolution and inter-rater reliability, restrict their application in the clinic setting (157, 158).

## Conclusions and Perspective

**Figure 5:**
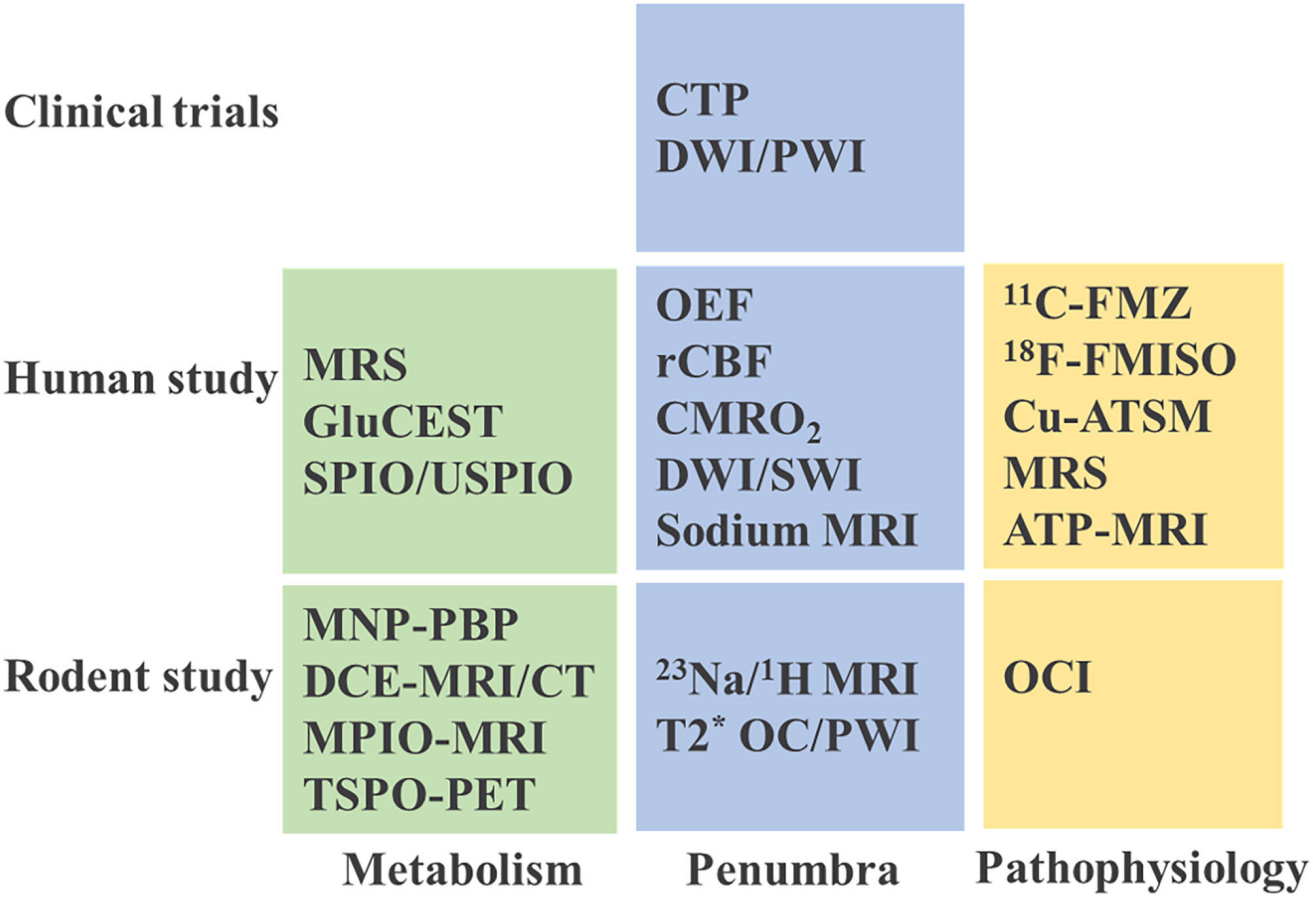
Current imaging practice in stroke studies. MRS, magnetic resonance spectroscopy; GluCEST, glutamate weighted chemical exchange saturation transfer; SPIO, superparamagnetic iron oxide; USPIO, ultrasmall superparamagnetic iron oxide; MNP-PBP, magnetic nanoparticle-P-selectin binding peptide; MRI, magnetic resonance imaging; DCE, dynamic contrast enhanced; CT, computed tomography; MPIO, microparticles of iron oxide; TSPO-PET, translocator protein-positron emission tomography; OEF, oxygen extraction fraction; rCBF, regional cerebral blood flow; CMRO_2_, cerebral metabolic rate of oxygen; DWI/SWI, diffusion weighted imaging/ susceptibility weighted imaging; ${\text{T}}_{2}^{*}$OC/PWI, ${\text{T}}_{2}^{*}$ oxygen challenge/perfusion-weighted imaging; ^11^C-FMZ, ^11^C-flumazenil; ^18^F-FMISO, ^18^F-Fluoromisonidazole; Cu-ATSM, copper-diacetyl-bis(N_4_-methylthiosemicarbazone); ATP, adenosine triphosphate; OCI, oxygen challenge imaging.

Reperfusion therapies are critically time dependent. The earlier treatment within time windows leads to more benefits. For the patients reached beyond the time windows, “tissue window” should be considered. Emerging evidence from recent clinical trials have recognized that tissue viability defined by the imaging modalities might be a more precise and reliable surrogate marker than time window (159). The newly issued guidelines have recommended evaluation of the penumbra or infarction by DWI, PWI, and CTP to expand eligibility for mechanical thrombectomy in the 6–24 h window after stroke onset. Compared to PDM, the novel imaging features on metabolism and pathophysiology, are more specific and sensitive to examine the salvageable tissue after stroke, and show great potential to define the therapeutic window. However, there are still challenges and controversies. Nonetheless, it needs to be said, that there is no reliable setting to determine the viability of brain tissue in the acute stage of stroke. Although visibility of such imaging setting as oxygen challenge MRI and DCE-MRI/CT for the detection of BBB leakage were tested in rodent studies, their usefulness for clinic have not been evaluated yet. Others, such as pH-weighted imaging and glutamate imaging, have only been validated in human studies, but all the same the utility of the concepts have not been evaluated in clinical trials yet (Figure 5). Though PDM tempts us to differentiate the potential salvageable tissue from infarction, unfortunately, the ideal imaging parameters and their accuracy remain elusive. Definitive validation is necessary in the future research and clinical trials. And because of a lack of standardization, the incorporation of the imaging modalities in clinical practice will be consequently limited. Future investigations on standardization of the imaging sequences and parameters to define tissue window are expected. Besides, the restricted attainability and long time needed for examination are burdens that limit PDM application for stroke patients. For instance, in Europe on average only 4% of the decision in regards of possible treatment will be based on MR perfusion and this number varied greatly from country to country (160). The time needed for an MRI perfusion scan is ~16 min. That is longer than 10 min, which are needed for CTP. However, it is possible to standardized stroke MRI protocols and reduce time in this way down to 10 min or even to 6 min by using echo planar imaging at 3 T (161). It is clear that with increased availability and standardization of the parameters, PDM could become the prime imaging technique able to precisely define the tissue window and identify patients eligible for endovascular thrombectomy.

## Author Contributions

JL, QH, YL, and XH wrote the manuscript. DL and JZ made suggestions for improvement. QM and LZ created the figures. AM edited the language. All authors read, revised, and approved the final manuscript.

## Funding

This research was supported by the National Natural Science Foundation of China No. 82071283 to QH, China Postdoctoral Science Foundation Grant No. 2018M632130 to JL, and the National Institutes of Health P01 NS082124 to JZ.

## Conflict of Interest

The authors declare that the research was conducted in the absence of any commercial or financial relationships that could be construed as a potential conflict of interest.

## Publisher's Note

All claims expressed in this article are solely those of the authors and do not necessarily represent those of their affiliated organizations, or those of the publisher, the editors and the reviewers. Any product that may be evaluated in this article, or claim that may be made by its manufacturer, is not guaranteed or endorsed by the publisher.

## Footnotes

^1^ https://seekingalpha.com/article/4263610-athersys-stem-cell-therapy-for-ischemic-stroke
